# How I do it: a standardized robotic workflow for image-guided stereo-electroencephalography: technical description and institutional experience

**DOI:** 10.1007/s00701-026-06937-w

**Published:** 2026-06-25

**Authors:** Pedro Roldan, Alvaro Bedoya Gómez, Gloria Cabrera, Jordi Rumià

**Affiliations:** 1 Department of Surgery, Faculty of Medicine, University of Barcelona, Barcelona, Spain; 2 Department of Neurosurgery, Hospital Clínic of Barcelona, C/Villarroel 170, 08036 Barcelona, Spain; 3 Department of Surgery and Surgical Specializations, University of Barcelona, Barcelona, Spain

**Keywords:** Stereo-electroencephalography (SEEG), Robotic neurosurgery, Epilepsy surgery, Stereotactic techniques, Depth electrodes, Robot-assisted electrode implantation

## Abstract

**Background:**

Stereo-electroencephalography (SEEG) is a key technique for the presurgical evaluation of drug-resistant focal epilepsy [2]. Robotic assistance facilitates precise multi-trajectory stereotactic implantation and allows workflow standardization [1, 3, 5, 9]. We describe a reproducible image-based robotic SEEG implantation protocol and report our institutional experience.

**Methods:**

An image-based robot-assisted SEEG technique using the Neuromate® system combined with intraoperative 3D imaging is presented. The workflow includes hypothesis-driven trajectory planning, alphabetical trajectory sequencing, laser-verified stereotactic registration, trajectory-specific skull thickness measurement with depth-controlled drilling, reducer-guided bolt placement, and intraoperative imaging verification. Institutional procedural data were reviewed to illustrate workflow performance and safety.

**Results:**

Sixty-eight patients underwent implantation of 952 SEEG depth electrodes (mean 14 electrodes per patient). Mean robotic implantation time was 198 min (SD 54.9; range 80–280), corresponding to 14.1 min per electrode. Mean stereotactic registration error was 1.7 mm. Three patients (4.4%) developed intracranial hemorrhage, with one symptomatic case (1.47%) requiring surgical evacuation. This event occurred during electrode removal rather than implantation. No hemorrhage occurred during robotic electrode insertion, and no electrode repositioning or reintervention was required.

**Conclusion:**

Image-based robot-assisted SEEG enables accurate and reproducible multi-trajectory implantation with efficient workflow and low complication rates. Structured trajectory planning and systematic intraoperative verification may improve procedural safety and standardization.

**Supplementary Information:**

The online version contains supplementary material available at 10.1007/s00701-026-06937-w.

## Relevant surgical anatomy

SEEG implantation requires precise planning of multiple cortical and subcortical trajectories [1, 2]. Unlike deep brain stimulation, accuracy depends not only on the final intracranial target but also on the geometric safety of each trajectory along its entire course.

Trajectory planning requires systematic evaluation of cortical entry points, gyral orientation, subcortical white matter pathways, ventricular relationships, and deep vascular structures. Entry points are preferably located at the crown of a gyrus to reduce the risk of vascular injury and improve mechanical stability during drilling and bolt fixation [1].

Cortical vascular anatomy must be carefully evaluated. Superficial cortical veins, bridging veins, and sulcal arteries should be avoided when selecting entry points. Vascular assessment is performed using contrast-enhanced T1-weighted MRI sequences fused with thin-slice CT imaging, allowing visualization of cortical veins, sulcal arteries, and deep vascular structures during trajectory planning [5, 9].

Subcortical trajectory planning must consider ventricular cavities, deep perforators, and major venous structures [2, 3]. Although orthogonal trajectories improve mechanical stability and reduce skiving at the bone interface, oblique trajectories are sometimes required for mesial temporal, insular, or opercular explorations [3].

For each trajectory, skull thickness measurement is essential to determine appropriate drilling depth and bolt length. In image-based robotic SEEG, even small stereotactic registration errors may propagate across multiple electrodes; therefore, precise anatomical planning is critical to ensure procedural safety and interpretability of electrophysiological recordings.

## Technique

### Preoperative multidisciplinary evaluation

**Fig. 1:**
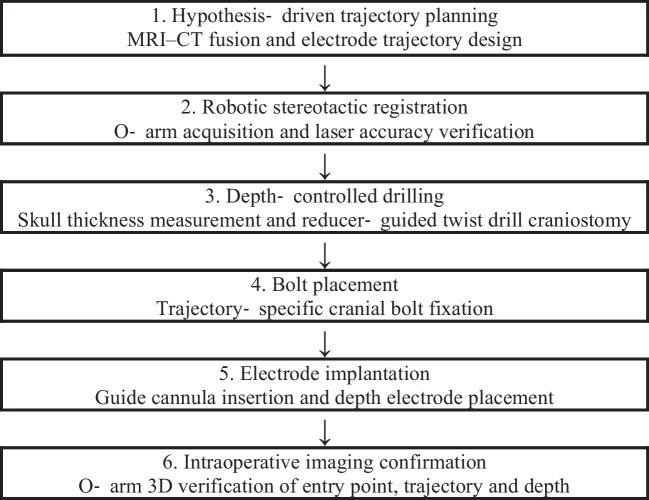
Image-based robotic SEEG implantation workflow. Schematic representation of the standardized robotic implantation protocol. The workflow includes hypothesis-driven trajectory planning, robotic stereotactic registration with intraoperative 3D imaging, reducer-guided depth-controlled drilling based on skull thickness measurement, cranial bolt placement, depth electrode insertion, and final intraoperative imaging verification

All cases are discussed in a comprehensive epilepsy conference involving epileptologists, epilepsy neurosurgeons, neuropsychologists, neuroradiologists, neuropsychiatrists, and nuclear medicine specialists [8] (Fig. 1).

**Fig. 2:**
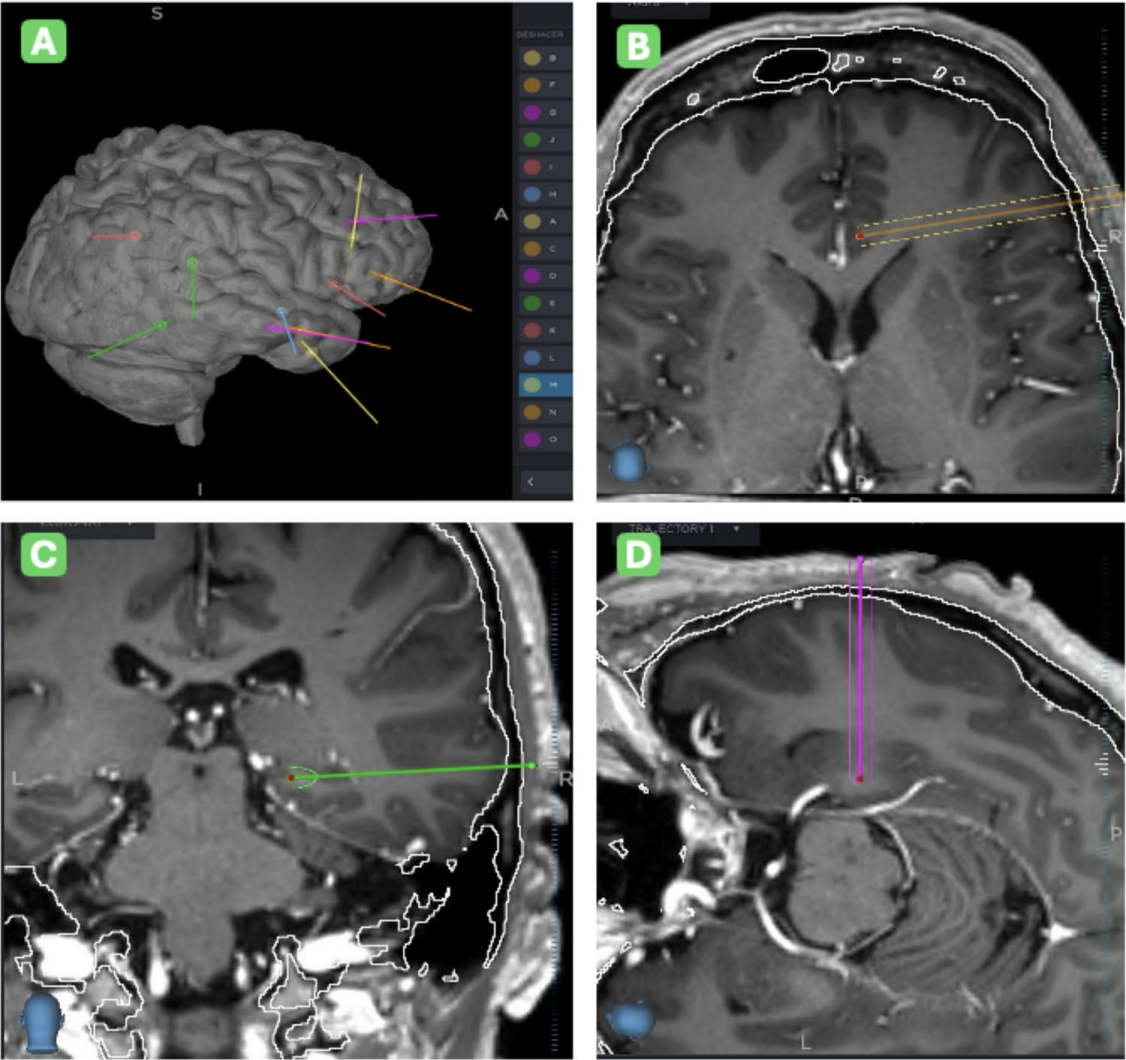
Preoperative trajectory planning for robot-assisted stereo-electroencephalography. **A** Three-dimensional cortical surface reconstruction illustrating planned electrode trajectories. **B** Axial MRI view demonstrating stereotactic trajectory planning toward deep cortical targets. **C** Coronal reconstruction showing trajectory orientation relative to ventricular structures. **D** Sagittal view illustrating orthogonal trajectory planning and target depth

Based on noninvasive data, a hypothesis-driven scheme of potential epileptogenic zones is defined [2, 8]. Electrode targets and orthogonal trajectories are then designed to sample cortical and subcortical regions relevant to the working hypothesis [8] (Fig. 2).

The total number of electrodes is established, and each electrode is assigned a sequential alphabetical designation (A–Z). The sequence follows a posterior-to-anterior and inferior-to-superior zigzag pattern. This structured order allows the robotic arm to execute movements in a reproducible geometric sequence, minimizing unnecessary repositioning.

**Fig. 3:**
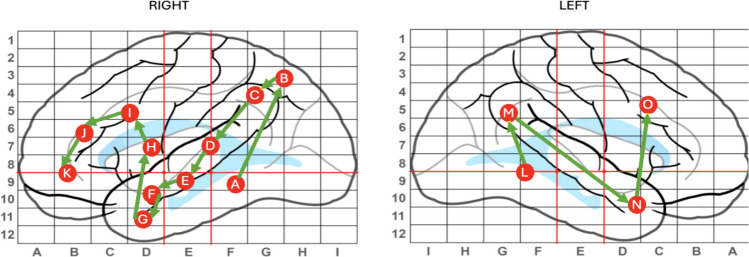
Schematic representation of electrode distribution and implantation sequence in robot-assisted SEEG. The diagram illustrates the predefined implantation strategy using alphabetical electrode designation and a posterior-to-anterior, inferior-to-superior zigzag sequence. The schematic demonstrates bilateral electrode distribution and the geometric order used to optimize robotic arm movement during multi-trajectory implantation

For bilateral cases, implantation begins in the hemisphere requiring the greater number of electrodes (Fig. 3).

**Fig. 4:**
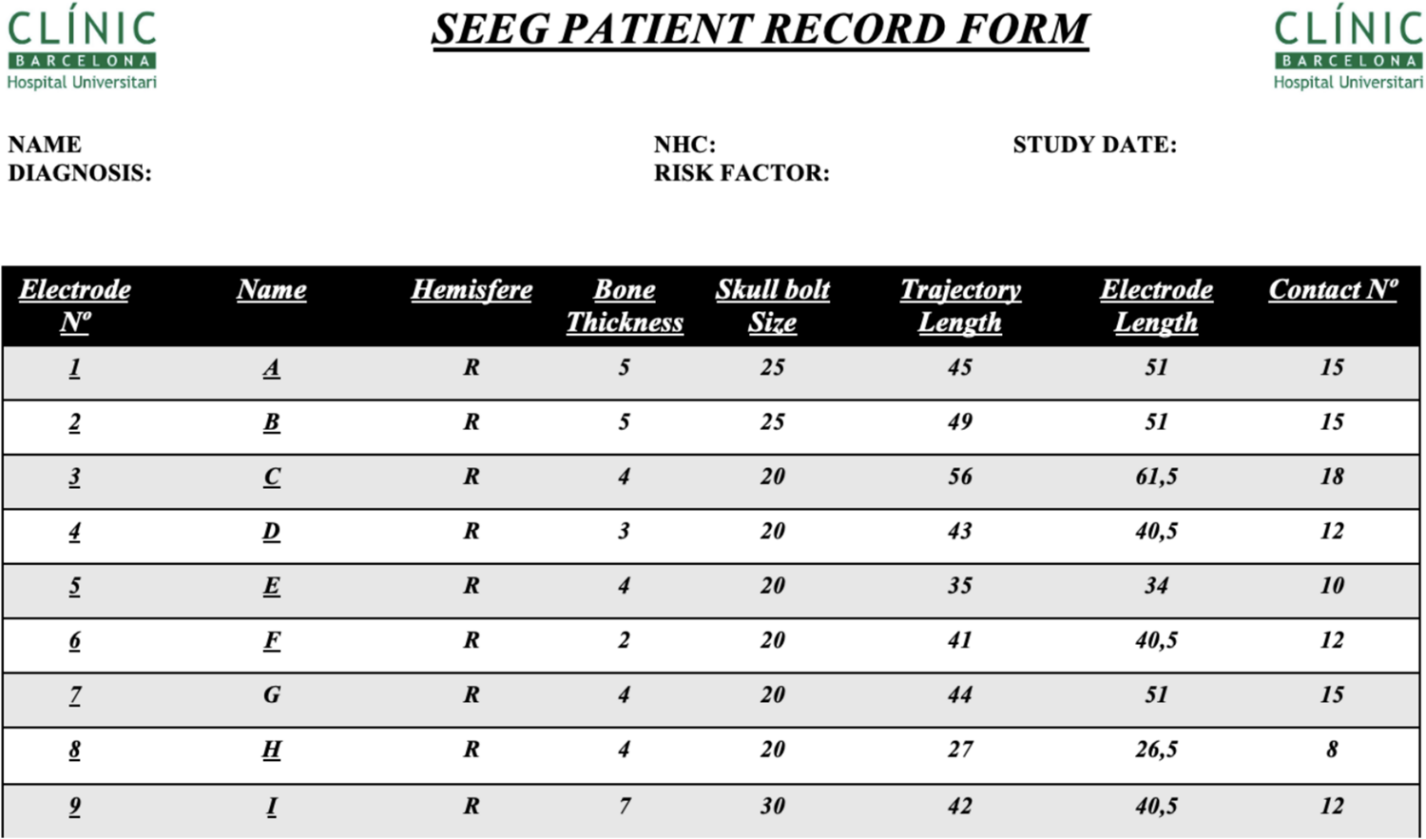
Institutional SEEG patient record form integrated into the implantation workflow. The form documents key trajectory parameters—including skull thickness, bolt size, trajectory length, electrode length, and number of contacts—and functions as a procedural safety checklist to verify technical variables before electrode implantation

Bone thickness, skull bolt length, trajectory measurements, electrode extension, and number of contacts are systematically documented in the institutional SEEG form. This form serves as a procedural safety checkpoint, enabling verification of these parameters before electrode implantation and reducing the risk of technical errors during SEEG placement (Fig. 4).

### Operating room setup and head fixation

The procedure is performed under general anesthesia.

While anesthesia is induced, the Leksell frame is assembled. Under general anesthesia, rigid head fixation is achieved using four pins. The frame is not used for stereotactic referencing but provides rigid coupling to the Neuromate® robotic base.

The patient is positioned supine with 5–10 degrees of trunk elevation to facilitate venous drainage and reduce the risk of pneumocephalus. The frame is secured to the robotic platform.

A frontal adhesive fiducial marker is placed on the scalp to allow robotic laser-based accuracy verification.

The Neuromate laser system is mounted on the robotic arm, followed by attachment of the Neurolocate verification module. The system is positioned adjacent to the fronto-parietal region to allow unobstructed trajectory execution.

### Robotic registration and accuracy verification

Intraoperative 3D imaging is acquired using an O-arm system. Images are fused with the preoperative planning dataset [6].

Fiducial recognition is performed, and system accuracy is quantified. Registration precision is expressed in millimetric values. Manual adjustment is performed if necessary [1, 3, 9].

A virtual safety sphere is defined within the robotic workstation. This sphere restricts robotic movement beyond predefined spatial limits, reducing the risk of unintended collisions.

Final stereotactic calibration is verified by executing a laser test trajectory toward the previously placed frontal fiducial marker. This step confirms concordance between planned and executed coordinates before electrode implantation begins.

The O-arm acquisition and parking positions are recorded to allow reproducible intraoperative imaging later in the procedure.

The patient and robotic arm are then prepared and draped in sterile fashion.

Although this workflow was implemented using the Neuromate® robotic system, the described procedural principles—including hypothesis-driven planning, depth-controlled drilling, and intraoperative imaging verification—are conceptually applicable to other robotic SEEG platforms.

### Electrode implantation

After sterile preparation and draping of both the cranial field and robotic arm, implantation begins according to the predefined alphabetical sequence.

The first trajectory selected corresponds to electrode “A,” typically the most posterior and inferior entry point in the zigzag sequence. The corresponding target and trajectory are activated within the Renishaw planning software.

A 2.5 mm reducer is mounted onto the robotic arm. The robotic arm is positioned automatically along the predefined stereotactic coordinates, aligning the reducer coaxially with the planned trajectory [1].

### Skin and soft tissue access

A sharp punch is advanced through the reducer to incise skin, subcutaneous tissue, and periosteum in a single linear tract. This step ensures coaxial alignment between soft tissue opening and the planned trajectory.

A monopolar coagulation probe is then introduced through the reducer to coagulate the soft tissue tract. This reduces bleeding and minimizes tissue drag during drilling.

### Bone drilling and depth control

A 2.5 mm drill bit is inserted through the reducer.

Before drilling, the thickness of the skull along the specific trajectory—previously measured during planning—is confirmed. Using a sterile ruler, the measured skull thickness is added from the base of the reducer, and a mechanical stop is placed on the drill bit to act as a depth limiter. This prevents inadvertent dural penetration during drilling [4].

A 2.5 mm twist drill craniostomy is performed under continuous irrigation. The drill stop ensures controlled bone perforation.

Following bone perforation, a coagulation probe is again introduced through the reducer to coagulate the exposed dura.

### Dural opening

The dura is coagulated and then sharply opened using a monopolar tip through the reducer. The opening is kept minimal and strictly coaxial to the trajectory to reduce cerebrospinal fluid (CSF) egress and minimize pneumocephalus.

Excessive dural opening is avoided to maintain intracranial pressure stability, particularly in multi-electrode implantations.

### Skull bolt placement

A skull bolt is inserted through the reducer. The bolt length is selected according to the measured skull thickness for that trajectory. The bolt is advanced until firmly secured within the calvarial bone, ensuring stable fixation without excessive compression.

### Guide insertion

A guide cannula with stylet, pre-measured according to the planned depth, is introduced through the bolt along the robotic trajectory.

The guide is advanced to the predefined depth corresponding to the intracranial target. Once in position, it is left in place for approximately 10–15 s. This pause may facilitate tract stabilization and theoretical microvascular tamponade.

The guide with stylet is then gently withdrawn in a controlled fashion.

### Electrode insertion and fixation

The corresponding depth electrode, previously labeled according to the alphabetical sequence, is introduced through the bolt along the established trajectory.

The electrode is advanced to the planned depth under robotic guidance.

After confirming correct insertion depth based on the stereotactic plan, the bolt cap is tightened to secure the electrode in position.

The distal externalized portion of the electrode (intended for connection to the SEEG recording system) is placed into an individual sterile bag positioned adjacent to the patient. Care is taken to avoid traction or torsion on the implanted electrode.

This entire sequence is repeated for each electrode following the predefined alphabetical order. The robotic arm repositions automatically between trajectories according to the planned zigzag geometric pattern.

**Fig. 5:**
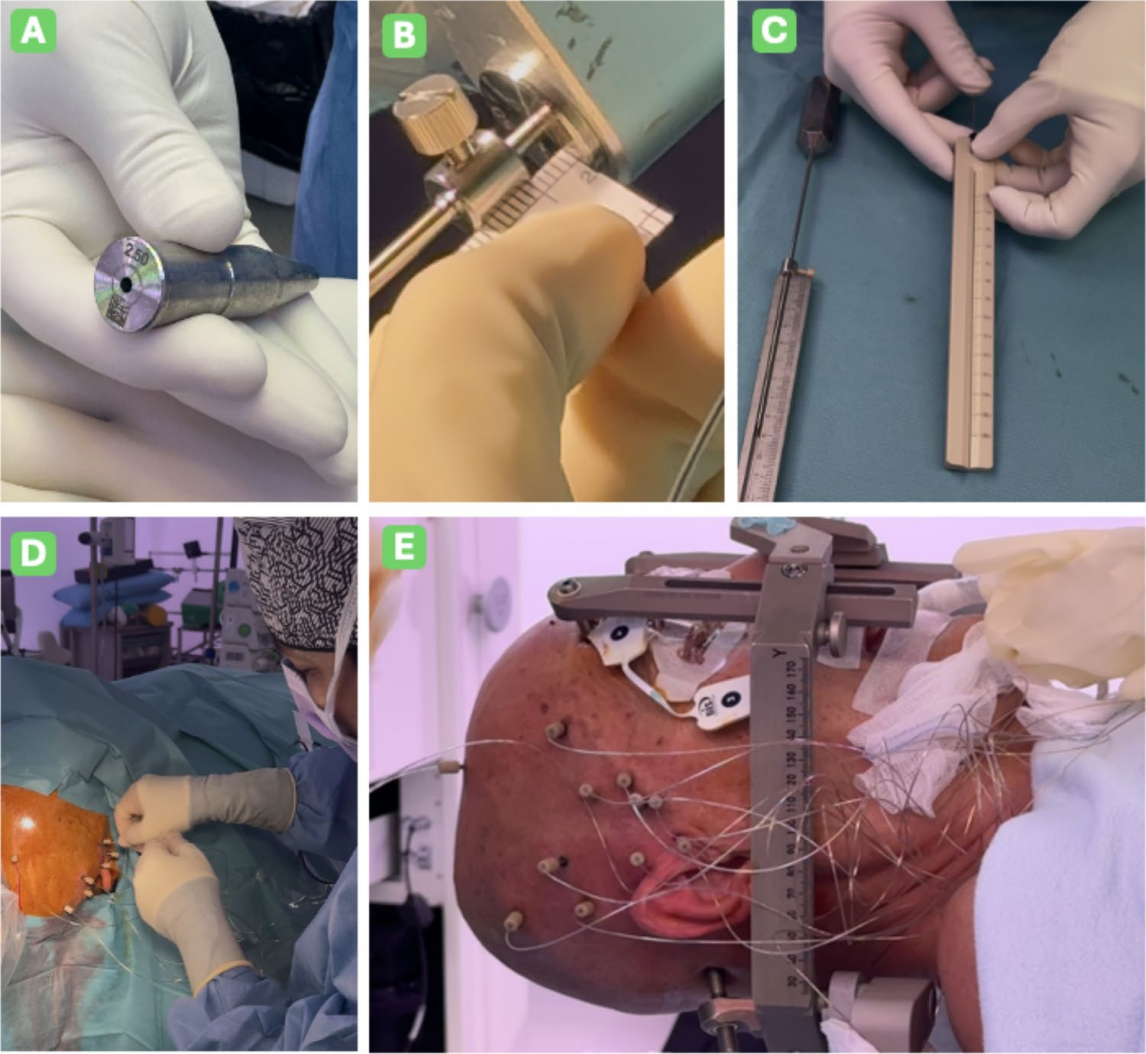
Intraoperative technical steps during robot-assisted SEEG implantation. **A** 2.5-mm reducer used for coaxial alignment of instruments along the planned stereotactic trajectory. **B** Measurement of drilling depth according to trajectory-specific skull thickness. **C** Adjustment of electrode length prior to insertion. **D** Intraoperative handling and connection of depth electrodes during implantation. **E** Final intraoperative view showing multiple implanted SEEG electrodes secured with cranial bolts

At completion of all implantations, the sterile bag containing the distal portions of the electrodes is sealed (Fig. 5).

### Intraoperative imaging and verification

After all electrodes are implanted and secured, intraoperative 3D imaging is acquired using the O-arm system.

Images are fused with the preoperative planning dataset to confirm entry point concordance, trajectory alignment, depth accuracy, spatial relationships between electrodes, and absence of immediate hemorrhagic complications [5, 8].

Each electrode is individually reviewed in multiplanar reconstruction [10].

Minor deviations are interpreted in relation to anatomical landmarks. If significant deviation is detected, revision can be performed during the same surgical session.

Once satisfactory positioning is confirmed, the robotic arm is disengaged.

### Completion of procedure and immediate postoperative care

After imaging confirmation, sterile drapes are removed.

The patient is transferred to the post-anesthesia care unit (PACU/URPA) for approximately six hours of neurological monitoring.

Continuous clinical assessment is performed to detect early neurological deficits or signs suggestive of intracranial hemorrhage.

Electrophysiological recording is initiated according to institutional epilepsy monitoring protocols after appropriate recovery.

The described workflow has been progressively standardized in our institution over the past years. To illustrate the procedural performance and safety of this technique, we reviewed our consecutive institutional experience with this image-based robot-assisted SEEG protocol using a descriptive analysis. The aim of this analysis was not to perform a comparative clinical study but to provide quantitative procedural metrics associated with the described workflow. Institutional review board approval was obtained according to institutional research regulations, and all procedures were performed as part of standard clinical care.

## Technical pearls

Several technical aspects contribute to the safety and reproducibility of image-based robot-assisted SEEG implantation.

Structured alphabetical trajectory sequencing. Electrodes are implanted following a predefined alphabetical order organized in a posterior-to-anterior and inferior-to-superior zigzag pattern. This structured sequence minimizes robotic arm repositioning and optimizes procedural workflow.

Trajectory-specific skull thickness measurement. For each electrode trajectory, skull thickness is measured during preoperative planning. A mechanical drill stop is placed on the drill bit according to this measurement, allowing controlled bone perforation and preventing inadvertent dural penetration.

Coaxial reducer-guided drilling and implantation. All procedural steps—including soft tissue access, drilling, bolt insertion, and electrode placement—are performed through the robotic reducer. This ensures strict coaxial alignment between the planned stereotactic trajectory and the surgical instruments.

Guide cannula dwell time. After advancement to the target depth, the guide cannula is left in position for approximately 10–15 s before withdrawal. This brief dwell time may promote tract stabilization and theoretical microvascular tamponade.

Laser-based registration verification. A robotic laser trajectory directed toward a frontal fiducial marker is used to verify stereotactic registration accuracy before electrode implantation begins.

Systematic intraoperative 3D imaging confirmation. Final electrode position is verified using intraoperative 3D imaging fused with the preoperative planning dataset, allowing confirmation of entry point concordance, trajectory alignment, and depth accuracy before completion of the procedure.

## Institutional experience

Over a 7-year period, 68 consecutive patients underwent image-based robot-assisted SEEG implantation at our institution. A total of 952 depth electrodes were implanted, corresponding to a mean of 14 electrodes per patient.

Mean robotic implantation time, defined as the interval from activation of the first trajectory to fixation of the final electrode, was 198 min (SD 54.9; range 80–280). The calculated mean implantation time per electrode was 14.1 min.

Mean stereotactic registration error was 1.7 mm. These findings are consistent with previously reported robotic SEEG series and meta-analyses evaluating procedural accuracy and complication rates [9].

Three patients (4.4%) developed intracranial hemorrhage. Only one case (1.47%) was symptomatic and required surgical evacuation. This event occurred during electrode removal rather than during implantation. No hemorrhagic complication occurred during robotic electrode insertion.

No electrode repositioning or surgical reintervention for inaccurate placement was required. The cumulative hemorrhage rate per implanted electrode was 0.31% (3/952).

These institutional findings support the safety, reproducibility, and mechanical stability of the described workflow. Appropriate patient selection remains essential to maximize diagnostic yield and procedural benefit.

The operative efficiency and complication profile observed in this series are consistent with previously reported robotic SEEG workflows.

## Indications

Image-based robot-assisted SEEG is indicated in patients with drug-resistant focal epilepsy requiring invasive evaluation for localization of the epileptogenic zone, particularly when noninvasive investigations provide insufficient or discordant localization data [6].

Candidates are selected following multidisciplinary evaluation and when noninvasive studies fail to provide sufficient concordant localization data.

The technique requires dedicated institutional infrastructure and a trained multidisciplinary team.

## Limitations

Image-based robot-assisted SEEG requires significant institutional investment in robotic platforms and intraoperative imaging systems.

The technique depends on high-quality image acquisition and precise registration. Registration errors may propagate across multiple electrodes [1].

Extremely oblique trajectories may challenge mechanical stability depending on skull curvature and bolt angulation limits.

Learning curve effects are present during early implementation phases [7].

The present report represents a single-center technical experience and does not include comparative analysis with frame-based or other robotic workflows.

## How to avoid complications

- Systematic avoidance of sulci and cortical veins during planning
- Mandatory skull thickness measurement for each trajectory
- Use of mechanical drill stop to prevent dural penetration
- Minimal dural opening to reduce CSF loss
- Sequential hemispheric strategy prioritizing the more affected side
- Guide cannula dwell time of 10–15 s before withdrawal
- Strict verification of robotic registration accuracy
- Routine intraoperative 3D imaging confirmation

## Key points

- Image-based robot-assisted SEEG enables reproducible multi-trajectory implantation.
- Alphabetical electrode sequencing optimizes robotic workflow.
- Skull thickness measurement is mandatory for safe drilling.
- Controlled dural opening minimizes pneumocephalus.
- Guide dwell time may reduce hemorrhagic risk.
- Intraoperative 3D imaging confirms spatial accuracy before completion.

## Supplementary Information

Below is the link to the electronic supplementary material.

ESM 1 Supplementary Material 1 (MP4 262 MB)

## Data Availability

No datasets were generated or analysed during the current study.

## Abbreviations

*SEEG*: Stereo-electroencephalography
*MRI*: Magnetic resonance imaging
*CT*: Computed tomography
*O-arm*: Intraoperative 3D imaging system
*CSF*: Cerebrospinal fluid
*FRE*: Fiducial registration error
*PACU*: Post-anesthesia care unit
